# Expression profile of LncRNA ANRIL, miR-186, miR-181a, and MTMR-3 in patients with preeclampsia

**DOI:** 10.1016/j.ncrna.2023.06.001

**Published:** 2023-06-07

**Authors:** Shymaa E. Ayoub, Olfat G. Shaker, Rehab Abdelhamid Aboshama, Mohamed K. Etman, Abeer A. Khalefa, Mohamed M. khamiss Abd elguaad, Othman M. Zaki, Doaa Y. Ali, Nada F. Hemeda, Amal Amin, Marwa A. Ali

**Affiliations:** a Department of Biochemistry and Molecular Biology, Faculty of Medicine, Fayoum University, Al Fayoum, Egypt; b Department of Biochemistry and Molecular Biology, Faculty of Medicine, Cairo University, Cairo, Egypt; c Department of Obstetrics and Gynecology, Faculty of Medicine, Fayoum University, Al Fayoum, Egypt; d Department of Physiology, Faculty of Medicine, Zagazig University, El Zagazig, Egypt; e Department of Physiology, Faculty of Medicine, Fayoum University, Al Fayoum, Egypt; f Department of Clinical Pathology, Faculty of Medicine, Damietta University, Damietta, Egypt; g Department of Clinical and Chemical Pathology, Faculty of Medicine, Fayoum University, Al Fayoum, Egypt; h Department of Genetics, Faculty of Agriculture, Fayoum University, Fayoum, Egypt; i Department of Medical Microbiology and Immunology, Faculty of Medicine, Fayoum University, Al Fayoum, Egypt

**Keywords:** Preeclampsia, LncRNA ANRIL, miR-186, miR-181a, MTMR-3

## Abstract

Preeclampsia (PE) is a leading cause of maternal and neonatal morbidity and mortality worldwide. Several studies demonstrated the role of lncRNAs and miRNAs in the pathogenesis of preeclampsia; the aim was to detect the expression profiles of serum LncRNA ANRIL, miR-186, miR-181a, and MTMR-3 in patients with preeclampsia. The study included 160 subjects divided into 80 subjects considered as a control group, 80 patients with preeclampsia. We found that there was a significant difference between the preeclampsia and control groups with up-regulation of miR-186 median (IQR) = 4, 29 (1.35–7.73) (P < 0.0001), miR-181a median (IQR) = 2.45 (0.83–6.52) (P = 0.028), and downregulation of lncRNA ANRIL median (IQR) = 0.35(0.28–0.528) (P < 0.0001), MTMR median (IQR) = 0.32(0.155–1.11), (P < 0.0001). ROC curve of lncRNA ANRIL, miR-186, miR-181a, and MTMR-3 in preeclampsia patients showing the roles of these markers in the diagnosis of preeclampsia. In conclusion, serum LncRNA ANRIL, miR-186, miR-181a, and MTMR-3 could be promising biomarkers in the diagnosis of preeclampsia.

## Abbreviations

Preeclampsia: PE
ANRIL: LncRNA antisense noncoding RNA in the INK4 locus
mTOR: mammalian target of rapamycin
GJB2: Gap junction beta-2 protein
MTMR3: Myotubularin-Related Protein 3
SPSS: Statistical Package for the Social Sciences
ROC curve: Receiver operating characteristic curve
PLD: phospholipase D
APAF1: apoptotic protease activating factor 1
cSCC: cutaneous squamous cell carcinoma cells
ATG5: Autophagy Related 5

## 1 Introduction

Preeclampsia (PE) is a pregnancy-related disorder that affects 4.6% of all pregnancies, all over the world and is one of the most common causes of maternal and neonatal mortality [1]. In early-onset preeclampsia, the process starts with poor placentation and vascularization that occurs by an insufficient trophoblastic invasion of the uterine arteries then the condition is aggravated by activation of the immune system [[2], [3], [4]]. During normal pregnancies, placental autophagy (the degradation of damaged or dysfunctional cellular components and the recycling of cellular constituents) prevents aggregation of proteins in the trophoblasts; these aggregations could cause abnormal development of the placenta by activation of apoptosis and cellular senescence resulting in telomere shortening or dysfunction [5]. Many recent studies demonstrate the role of non-coding RNA (lncRNAs and miRNAs) in the regulation of autophagy in vitro and in vivo [6].

ANRIL (LncRNA antisense noncoding RNA in the INK4 locus), is transcribed from the INK4b-ARF-INK4a locus [7]. ANRIL is responsible for the function of phospholipase D (PLD) inhibitor which destabilizes mTOR signaling pathway scausing activation of autophagy [8].

MiRNAs regulate autophagy by changing the levels of different proteins responsible for several steps of the autophagy process. MiR-181a was found to be a modulator of autophagy in gastric cancer cells by downregulation of Myotubularin-related protein 3 (MTMR3) [9]. MTMR3, a phosphatidylinositol 3-phosphate (PI3P) phosphatase, is one of the principal genes involved in autophagy pathway regulation [10,11]. MTMR3 has a multi-model function in the regulation of autophagy: inhibition of mTORC1 and reduction in local PI3P level [12].

MiR-186 has the potential to inhibit glioma-conditioned human cerebral microvascular endothelial cell autophagy by targeting Atg7 and Beclin1 [13].

Recent research reported the interaction between lncRNA ANRIL and microRNAs (181a and 186) in numerous diseases, in particular when it comes to cancer. ANRIL through inhibition of miR-181a promotes proliferation of laryngeal carcinoma [14]. ANRIL by sponging miR-186 could endorse cervical cancer development and enhance risk stratification and bad prognosis in multiple myeloma [15].

However, no previous study has investigated the relationship between ANRIL and miR-181a, and miR-186 in preeclampsia. Thus, we designed this case-control study to detect and correlate the expression profiles of lncRNA ANRIL, its target miRNAs (miR-186, miR-181a), and MTMR-3 (target for miR181a) as autophagy-regulated genes in pregnant women with and without preeclampsia and also relate their levels to relation to patients' clinical and biochemical investigations.

## 2 Materials and methods

### 2.1 Subjects

The study was conducted on 160 subjects divided into 80 normally pregnant women as control subjects and another 80 women with preeclampsia. Preeclampsia was diagnosed by: systolic blood pressure >140 mmHg and/or diastolic blood pressure>90 mmHg, accompanied by a urinary protein level that was >0.3 g in a 24 h urine collection [16]. Patients were enrolled from the Department of Obstetrics and Gynecology, Fayoum University Hospital, Egypt. The study was approved by the Ethical Committee of the Faculty of Medicine (R256), Fayoum University.

### 2.2 Samples collection

8 ml of blood was taken and collected in 3 tubes one of them for complete blood count, the 2nd for estimation of prothrombin time, and the 3rd tube for serum separation which used for the measurement of molecular and serological analysis.

### 2.3 RNA extraction

By using (Qiagen, Valencia, CA, USA) extraction kits and as prescribed by the data supplied by the manufacturer, 200 μl of serum was added to 1000 μl QIAzol lysis reagent and then incubate for 5 min followed by 200 μl chloroform to each sample followed by centrifugation for 15 min at 12,000×*g*. RNA quality was estimated by NanoDrop2000.

### 2.4 Reverse transcription and RT-PCR reactions

Regarding miR-181a and miR-186, the total volume was 25 μL per reaction using the miScript miRNA PCR primers assay (Qiagen, Valencia, CA). SNORD 68 was used as an internal control for miR-181a and miR-186. Regards to LncRNA ANRIL 20 μL was used.GADPH was used as an internal control for LncRNA ANRIL. The qRT-PCR was programmed as follows: Heating at 95 °C for 15 min, followed by 50 cycles of 94.0° for 15 s, 60.0° for 30 s, and 72 °C for 30 s for denaturation, annealing, and extension of DNA. The relative expression of RNAs was calculated by the 2^-ΔΔCt^ method [17]

Regarding MTMR3 mRNA, 100 ng of total RNA was used for reverse transcription

using a High-Capacity cDNA Reverse Transcriptase kit (Applied Biosystems, USA) with a total volume of 20 μl, GAPDH was used as internal control, and a Maxima SYBR Green PCR kit (Thermo, USA), The primer sequences were as follows: MTMR3-forward 5′ AGCAGAGTGGGCTCAGTGTT-3′, MTMR3-reverse 5′-ACTGTCCACGTTTGGT -CCTC-3′, Real-time PCR was performed in 20 μl reaction mixtures using Rotor-Gene Q System (Qiagen).

### 2.5 Statistical analysis

SPSS software version 22 (SPSS Inc, USA) was used. The mean, median, standard deviation, and range were calculated for quantitative data, the Mann-Whitney-U test was used if the variables were not normally distributed, meanwhile, the Independent-t-test was used if the variables were normally distributed in the comparison between different groups. Qualitative data were presented as numbers and percentages, and chi-square (χ2) was used as a test of significance. Spearman correlation was done to detect the relation of miR- 186, miR-181a, LncRNA ANRIL, and MTMR3 with study parameters. ROC curve was used to determine the cut-off points, significance was adopted at P ≤ 0.05.

## 3 Results

### 3.1 Demographic and biochemical characteristics of the study groups

Table 1, Table 2 showed that there was a statistically significant difference between patients and control with regard to systolic and diastolic blood pressure, mean arterial pressure (MAP), Fetal birth weight (P < 0.0001) for each, the albumin (P = 0.002), Aspartate transaminase (AST) (P = 0.003), direct bilirubin (P = 0.020) and C-reactive protein (CRP) (P = 0.005).

**Table 1:** Demographic and clinical characteristics of the study groups.

Variable	Cases (N = 80)		Control (N = 80)		
	Mean	±SD	Mean	±SD	P value
Age	29.73	6.874	31.00	6.601	0.403
BMI	31.20	5.297	32.36	4.732	0.304
Systolic blood pressure	164.39	17.578	116.79	12.952	**<0.001**
Diastolic blood pressure	108.29	11.046	72.31	7.057	**<0.001**
MAP	134.44	12.5	94.55	8.309	**<0.001**
Gestational week	36.87	2.053	37.60	1.543	0.075
Fetal birth weight	2.841	.4929	3.279	.3861	**<0.001**
	**Median**	**IQR**	**Median**	**IQR**	
Gravidity	2	0–3.5	1	0–4	0.953
Parity	2	0–3	1	0–3	0.667
Abortion	0	0–1	0	0–1	0.638

BMI, Body mass index; MAP, Mean arterial pressure; IQR, Interquartile range.

**Table 2:** Biochemical characteristics of the study groups.

Variable	Cases (N = 80)		Control (N = 80)		
	Mean	±SD	Mean	±SD	P value
HB% gl/dl	10.74	1.198	10.74	1.251	0.995
HCT	33.41	2.451	33.34	2.713	0.899
RBCS	4.092	.3421	4.151	.2781	0.403
MCH	26.73	3.240	27.08	3.519	0.672
MCHC	33.33	8.744	32.24	2.854	0.472
WBC	8232.93	3026.954	8123.08	2607.122	0.863
Platelets	2.87*10^5^	95592.8	3.04*10^5^	80788.5	0.381
ALK.PH (IU/L)	74.85	5.977	73.82	2.126	0.311
Albumin (mg/dl)	3.141	.4838	3.518	.5707	**0.002**
Urea (mg/dl)	25.24	9.962	26.23	10.202	0.663
FBS (mg/dl)	81.24	13.266	82.85	13.844	0.599
GLU PP (mg/dl)	118.29	15.222	115.82	13.964	0.452
PT	12.62	.696	12.66	.804	0.825
PC	105.41	22.488	106.10	19.910	0.885
PTT	33.46	6.000	33.26	5.500	0.873
	**Median**	**IQR**	**Median**	**IQR**	
INR	1	0.9–1	1	0.9–1	0.380
AST (IU/L)	24	14–34	14	11–25	**0.003**
ALT (IU/L)	19	15–31	17	10–25	0.105
T.Bilirubin(mg/dl)	0.5	0.3–0.705	.0.7	0.4–0.8	0.247
D.Bilirubin(mg/dl)	.07	.05–0.1	0.1	0.07–0.2	**0.020**
Indirect Bilirubin (mg/dl)	.40	.20–0.655	0.5	0.29–0.650	0.537
AST	24	14–34	14	11–25	**0.003**
CRP	27	5–57.5	6	3–30	**0.005**

HB gl/dl, Hemoglobin; HCT, Hematocrit; RBCS, Red blood cells; MCH, Mean cell hemoglobin; MCHC, Mean cell hemoglobin concentration; WBC, White blood cell; ALP, Alkaline phosphatase; FBS, Fasting blood sugar; GLU PP, 2-h glucose postprandial; PT, Prothrombin time; PC, Prothrombin concentration; T.Bilirubin, Total bilirubin; D.Bilirubin, Direct bilirubin; CRP, C- Reactive protein; IQR, Interquartile range.

### 3.2 The relative expression level of serum LncRNA ANRIL, miR-186, miR-181a, and MTMR-3

There was a significant difference between the patient and control groups regarding the median of the relative expression level of lncRNA ANRIL 0.35(0.28–0.528), miR-186 4, 29(1.35–7.73), MTMR 0.32(0.155–1.11), (P < 0.0001) for each and miR-181a 2.45 (0.83–6.52) (P = 0.028) (Table 3).

**Table 3:** The relative expression level of serum LncRNA ANRIL, miR-186, miR-181a, and MTMR-3.

Parameter	Median	(IQR)	P value
**LncRNA ANRIL**	0.35	(0.28–0.528)	**<0.001**
**miR-181a**	2.45	(0.83–6.52)	**0.028**
**miR-186**	4,29	(1.35–7.73)	**<0.001**
**MTMR-3**	0.32	(0.155–1.11)	**<0.001**

Fold change values show target genes expressions compared to controls, as determined using 2^-ΔΔCt^. Levels of control fold change are equal to 1.

### 3.3 Spearman correlation of lncRNA ANRIL, miR-186, miR-181a, and MTMR-3 with study parameters

Results showed that there was a negative significant correlation between the expression level of lncRNA ANRIL and MAP (*r* = - 0.311, P = 0.048), and positive with albumin (*r* = −0.355, P = 0.023) and FBS (*r* = -0. 353, P = 0. 024). Besides there was a negative significant correlation between the expression level of miR-181a with age (*r* = - 0.315, P = 0.045), and a positive significant correlation between MTMR-3 with BMI (*r* = 0.373, P = 0.016), Gravity (*r* = 0.436, P = 0.004), parity (*r* = 0.349, P = 0.025), FBS (*r* = 0.418, P = 0.007) (Table 4).

**Table 4:** Spearman correlation of LncRNA ANRIL, miR-186, miR-181a and MTMR-3 with study parameters.

		ANRIL	miR-181a	miR-186	MTMR-3
Age	r	−.067-	−.315*	−.168-	.133
	p	.677	**.045**	.292	.406
BMI	r	−.069-	−.121-	−.181-	.373*
	p	.666	.450	.258	**.016**
Gravity	r	−.086-	−.119-	.285	.436**
	p	.593	.460	.071	**.004**
Parity	r	−.046-	−.160-	.237	.349*
	p	.776	.317	.137	**.025**
Abortion	r	−.198-	−.063-	.061	.293
	p	.215	.697	.704	.063
MAP	r	−.311*	.173	.025	.006
	p	**.048**	.281	.875	.968
G. week	r	.133	.178	−.022-	−.124-
	p	.408	.264	.892	.441
Fetal birth weight	r	.170	−.222-	−.038-	.058
	p	.287	.163	.813	.719
HB gl/dl	r	.026	.120	.193	.189
	p	.871	.453	.228	.236
HCT	r	−.074-	.054	.235	.063
	p	.646	.737	.139	.695
RBCS	r	−.003-	−.017-	.079	.126
	p	.987	.915	.625	.433
MCH	r	−.008-	.078	.176	.129
	p	.958	.626	.270	.421
MCHC	r	.079	.211	.051	−.252-
	p	.625	.186	.750	.111
WBC	r	.292	−.256-	−.085-	.247
	p	.064	.107	.598	.119
Platelets	r	−.147-	.009	.022	.097
	p	.358	.956	.893	.545
AST(IU/L)	r	.216	.168	−.134-	−.301-
	p	.174	.293	.404	.056
ALT(IU/L)	r	.018	.298	.063	−.104-
	p	.912	.058	.697	.518
Albumin(mg/dl)	r	.355*	−.177-	.053	−.001-
	p	**.023**	.269	.743	.994
Total bilirubin(mg/dl)	r	−.062-	−.127-	.041	.118
	p	.702	.430	.799	.463
Direct B(mg/dl)	r	−.036-	.187	.220	.017
	p	.823	.242	.166	.917
Indirect B (mg/dl)	r	−.090-	−.169-	−.008-	.202
	p	.576	.291	.962	.206
ALK.PH(IU/L)	r	−.066-	.110	−.206-	−.224-
	p	.683	.492	.197	.159
CRP	r	−.161-	.219	−.089-	−.008-
	p	.313	.169	.581	.960
RBCs urine	r	.162	−.167-	−.232-	−.077-
	p	.313	.298	.144	.632
Pus urine	r	.124	−.097-	−.081-	.035
	p	.441	.547	.614	.826
Urea(mg/dl)	r	−.073-	−.158-	.072	.117
	p	.649	.324	.657	.467
FBS (mg/dl)	r	.353*	−.228-	−.013-	.418**
	p	**.024**	.151	.933	**.007**
GLU PP (mg/dl)	r	.141	−.240-	−.103-	.090
	p	.379	.131	.523	.576
PT	r	.251	−.104-	−.270-	.125
	p	.113	.519	.088	.435
PC	r	.134	.169	.000	.176
	p	.404	.291	1.000	.272
PTT	r	−.042-	−.248-	.043	−.084-
	p	.792	.119	.788	.603
INR	r	.103	.045	−.003-	.086
	p	.520	.780	.986	.594

BMI, Body mass index; MAP, Mean arterial pressure HB gl/dl, Hemoglobin; HCT, Hematocrit; RBCS, Red blood cells; MCH, Mean cell hemoglobin; MCHC, Mean cell hemoglobin concentration; WBC, White blood cell; ALP, Alkaline phosphatase; FBS, Fasting blood sugar; GLU PP, 2-h glucose postprandial; PT, Prothrombin time; PC, Prothrombin concentration; PTT, partial thromboplastin time; INR, International normalized ratio; T.Bilirubin, Total bilirubin; D.Bilirubin, Direct bilirubin; CRP, C-Reactive protein; IQR, Interquartile range.

### 3.4 Spearman correlation between lncRNA ANRIL, miR-186, miR-181a, and MTMR-3

Results showed that there was a negative significant correlation between the expression level of LNC ANRIL and miR-181a (*r* = −0.355, P = 0.023), miR-186 (*r* = -0.335, P = 0.032). As regards miR-186 there was a positive significant correlation with MTMR-3 (*r* = 0.320, P = 0.041). (Table 5).

**Table 5:** Spearman correlation between LncRNA ANRIL, miR-186, miR-181a and MTMR-3.

		ANRIL	miR-181a	miR-186	MTMR-3
**ANRIL**	**r**	1.000	−.355*	−.335*	.060
	**p**	.	**.023**	**.032**	.709
**miR-181a**	**r**	−.355*	1.000	.223	−.274-
	**p**	**.023**	.	.161	.083
**miR-186**	**r**	−.335*	.223	1.000	.320*
	**p**	**.032**	.161	.	**.041**
**MTMR-3**	**r**	.060	−.274-	.320*	1.000
	**p**	.709	.083	**.041**	.

### 3.5 Assessment of reliability of Sensitivity and Specificity of lncRNA ANRIL, miR-186, miR-181a, and MTMR-3 by ROC curve analysis

**Fig. 1:**
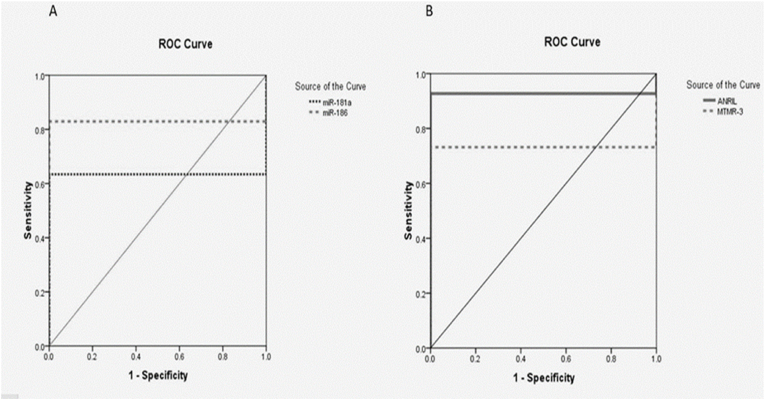
Receiver operating characteristic of Sensitivity and Specificity of LncRNA ANRIL, miR-186, miR-181a, and MTMR-3.

Figure (1) illustrates the Receiver operating characteristic **(**ROC curve) of lncRNA ANRIL, miR-186, miR-181a, and MTMR-3 in preeclampsia patients, showing their diagnostic value as predictors in differentiating between preeclampsia patients and healthy control. LncRNA ANRIL; AUC = 0. 927, P < 0.001, cut off point 0.935, sensitivity 92.7%, specificity 100.0%. miR-186; AUC = 0. 829, P < 0.0001, cut off point 1. 13, sensitivity 82.9%, specificity 100.0%. miR-181a; AUC = 0. 634, P = 0.034, cut off point 1.01, sensitivity 63.4%, specificity 100.0% and MTMR-3; AUC = 0. 732, P < 0.001, cut off point 0.996, sensitivity 73%, specificity100%

## 4 Discussion

Preeclampsia (PE) is a pregnancy-related disorder that can seriously threaten the safety of the mother and infant throughout the perinatal period [1], Autophagy is an important intrinsic process responsible for the turnover of old cellular proteins and organelles. and it is the principal process for adequate trophoblast invasion and normal placentation [6]. Recently, non-coding RNAs, such as long non-coding RNAs (lncRNAs) and miRNAs were demonstrated to regulate cell autophagy in vitro and in vivo [6]. The initial objective of our study is to evaluate for the first time the expression profiles of lncRNA ANRIL, its target miRNAs (miR-186, miR-181a), and MTMR-3 (target gene for miR-181a) in preeclampsia patients. Up and down-regulation of specific lncRNAs and microRNA could have a role in the PE pathogenesis by interrupting normal trophoblast proliferation, invasion, and apoptosis [[18], [19], [20], [21]].

As for the answer to the principle question of the study, we found that the relative expression *of* lncRNA ANRIL was significantly decreased by 0.35 (0.28–0.528), As regards miR-181a and MTMR-3, The relative expression level of miR-181a was up regulated 2.45 (0.83–6.52) (P = 0.028) and downregulation of the level of MTMR 0.32 (0.155–1.11). Meanwhile, the relative expression level of miR-186 was upregulated in preeclampsia patients 4, 29 (1.35–7.73).

The role of LncRNA ANRIL in autophagy was illustrated by ***Kang et al*** who reported that the increased expression level of antisense noncoding RNA in the INK4 locus (ANRIL) directly inhibited PLD (phospholipase D) and led to the induction of autophagy [8].

Recent research reported a negative interaction between LncRNA ANRIL and microRNAs (181a and 186) in numerous diseases, in particular when it comes to cancer [14,15]. These microRNAs are directly related to autophagy [13,22]. So ANRIL could modulate autophagy either in a direct way or indirectly by regulating autophagy-related microRNAs (181a and 186), a negative significant correlation between the expression level of lncRNA ANRIL with miR-181a (*r* = −0.355, P = 0.023) or with miR-186 (*r* = −0.335, P = 0.032) was found enforced this link.

MiR-181a was found to target many genes in the autophagy pathway; one of them is Atg5 which is a target of miR-181a in suppressing the autophagy of tumor cells. Atg5 is a gene that encodes autophagy protein 5, which is necessary for autophagosome elongation in autophagy [23]. Another study found that miR-181a targeting high-mobility group box 1 protein gene (HMGB1); ***Wang et al*** reported that ANRIL exacerbates the resistance of the chemotherapeutic drug gemcitabine in pancreatic cancer through inhibition of miR-181a thus activates HMGB1-induced autophagy [24]. The most recent gene is MTMR3; **Lin et al** found that miR-181a can inhibit autophagy in AGS gastric cancer cells by downregulating MTMR3 [9]. Also, **Senousy et al** found a link between MTMR3 rs12537 at miR-181a binding site with rheumatoid arthritis and systemic lupus erythematosus [25].

This study focused on MTMR3 as a principal gene in autophagy that linked miR-181a to PE.

Myotubularin-related protein 3 (MTMR3). MTMR3 has been reported to have opposite functions in the regulation of autophagy; a) MTMR3 inhibits mTORC1. MTORC1 controls every stage of the autophagy procedure; it prevents initiation and autophagosome formation by inactivating the autophagy regulatory complex (ULK1,Atg13and FIP200). mTORC1 also interferes with autophagy nucleation by targeting components of the class III PI3K complex I (PI3KC3-CI). In recent years, mTORC1 has been found regulate the autophagosome elongation. Furthermore, mTORC1 regulates autophagosome-lysosome fusion and autophagic flux termination via lysosomal tubulation. Thus, inhibition of mTORC1 by MTMR3 induces autophagy [26,27].MTMR3 has inositol lipid 3 phosphatase activity that dephosphorylates PI3P forming phosphatidyl inositol. MTMR3 reduces local phosphatidylinositol 3 phosphate (PI3P). PI3P is a lipid mediator of membrane trafficking and signaling that also has a role in autophagy initiation and formation of autophagosome. Thus, MTMR3 reduction was shown to promote autophagosome formation, however upregulation of MTMR3 resulted in smaller autophagosomes, which disabled autophagy [28].

These functions have opposing effects on autophagic flux. As a result, we concluded that MTMR3 regulates autophagy in multiple ways and that more research is needed to determine the precise significance of MTMR3in autophagy.

MTMR3 was found to be a direct target of miR-181a hence linking this miRNA to autophagy [9]. However, we did not find a correlation between serum miR-181a and MTMR3 expression in the PE group. ***Senousy et al*** reported a significant negative correlation between miR-181a and MTMR3 in SLE patients but not in rheumatoid arthritis patients [25]. The possible explanation for this unexpected finding is that MTMR3 is regulated by variables other than miR-181a that make the regulation of MTMR3 more complex mechanisms. The results showed a borderline significant positive correlation between MTMR3 and miR-186 for further review.

The relation detected between miR-186 and autophagy was controversial, previous two studies showed that miR-186 inhibits autophagy through decreased expression of Atg7 and Beclin1 [13] or by targeting inhibition of ATG14 [29] which are autophagy-induced genes. However, the most recent study demonstrated that miR-186 could induce autophagy by inhibiting the TLR4/MAPKs/NF-κB pathway [30].

The current study findings demonstrated a negative significant correlation between the expression level of lncRNA ANRIL and miR-186 (*r* = −0.335, P = 0.032). This could be explained by that lncRNAs and miRNAs share a complementary pairing sequence at 3′-UTR, allowing molecular-level binding and counteraction which modulates various physiological and pathological activities [31]. By using the bioinformatics program, ***Zhang et al*** [32] demonstrated the targeting of miR-186 with seven complementary binding sites for ANRIL. Besides they showed that miR-186 could reverse the function of ANRIL in cervical cancer cells.

Also, the results showed that there was a negative significant correlation between the expression level of miR-181a with LncRNA ANRIL (*r* = -0.355, P = 0.023). A study done by **Wang et al** examined the role of the LncRNA-ANRIL/miR-181//HMGB1 axis in regulating the autophagy of pancreatic cancer cells [24]**.** Also, ***Ying et al*** investigated the mechanism of lncRNA-ANRIL/miR-181b in autophagy of the cardiac cells in mice with uremia by targeting ATG5 [33]

***Lin et al*** found a link between miR-181a and autophagy was demonstrated through its direct target gene, myotubularin-related protein 3 (MTMR3) [34]; overexpression of miR181a depresses MTMR3 and vice versa.

From the abovementioned findings, we suppose that the four target genes are all linked to a cascade of events that end in inhibition of autophagy; for more details, we suggested that decreased LncRNA ANRIL stimulate upregulating miR-186 and miR-181a, thus, in turn, increased miR181a decreased MTMR3 resulting in suppressing of autophagy which is a beneficial physiological process responsible for the degradation of damaged or dysfunctional cellular components and the recycling of cellular constituents thus preventing protein aggregation in trophoblasts and placental malfunction anomalies.

The diagnostic significance of the lncRNA ANRIL, miR-186, miR181a, and MTMR3 as predictors of discriminating between preeclampsia patients and controls were investigated using ROC curve analysis. Four target biomarkers demonstrated great specificity (100% for all) and good sensitivity at cut-off points of .935, 1.13, 1.01, and 0.966 for ANRIL, miR-186, miR-181a, and MTMR3 respectively (92.7%, 82.9% 63.4%, and 73% for respectively). LncRNA ANRIL, miR-186, miR181a, and MTMR3 had AUCs of 0.927 and 0.829, 0.634, and 0,732 respectively.

The results presented in this featured article sought to identify ANRIL, miR-186, miR181a, and MTMR3 as new diagnostic biomarkers of preeclampsia with therapeutic potential. Further research is needed to confirm our findings and to overcome the current study's limitations, which involve (1) a relatively small sample size and (2) a deficient scientific functional publication demonstrating the key roles or molecular basis of target genes in the pathogenesis of preeclampsia. Larger-scale investigations that investigate the precise roles of these genes in the disease pathogenesis are necessary.

## 5 Conclusion

Serum lncRNA ANRIL, miR-186, miR-181a, and MTMR-3 could be used as potential biomarkers for the diagnosis of preeclampsia that may be used as therapeutic targets.

## Institutional review board statement

The study was approved by the Ethical Committee of the Faculty of Medicine (R256), Fayoum University. This study is consistent with the Declaration of Helsinki.

## Informed consent statement

Informed consent was obtained from all subjects involved in the study.

## Data availability statement

All relevant data are included in the article.

## Funding information

No financial support is relevant to this study.

## CRediT authorship contribution statement

**Shymaa E. Ayoub:** Conceptualization, Methodology, Validation, Data curation, Supervision, Project administration, Writing – original draft, Writing – review & editing. **Olfat G. Shaker:** Conceptualization, Formal analysis, Supervision. **Rehab Abdelhamid Aboshama:** Formal analysis, Investigation, Resources, Data curation, Writing – original draft. **Mohamed K. Etman:** Investigation, Resources, Writing – original draft. **Abeer A. Khalefa:** Software, Data curation, Writing – original draft. **Mohamed M. khamiss Abd elguaad:** Software, Data curation, Visualization, Methodology, Writing – original draft. **Othman M. Zaki:** Methodology, Investigation, Writing – original draft. **Doaa Y. Ali:** Methodology, Investigation, Writing – review & editing. **Nada F. Hemeda:** Methodology, Visualization, Writing – original draft. **Amal Amin:** Methodology, Visualization, Writing – original draft, Writing – review & editing. **Marwa A. Ali:** Conceptualization, Methodology, Validation, Resources, Writing – original draft, Writing – review & editing, Supervision, Project administration, Funding acquisition.

## Declaration of competing interest

No conflict of interest.

## References

[1] Abalos E., Cuesta C., Grosso A.L., Chou D., Say L. (2013). Global and regional estimates of preeclampsia and eclampsia: a systematic review. Eur. J. Obstet. Gynecol. Reprod. Biol..

[2] Ali S.M., Khalil R.A. (2015). Genetic, immune, and vasoactive factors in the vascular dysfunction associated with hypertension in pregnancy. Expert Opin. Ther. Targets.

[3] Kintiraki E., Papakatsika S., Kotronis G., Goulis D., Kotsis V. (2015). Pregnancy-Induced hypertension. Hormones (Basel).

[4] Amaral L., Wallace K., Owens M., Lamarca B. (2017). Pathophysiology and current clinical management of preeclampsia. Curr. Hypertens. Rep..

[5] Cox L.S., Redman C. (2017). The role of cellular senescence in ageing of the placenta. Placenta.

[6] Choudhry H., Harris A.L., McIntyre A. (2016). The tumour hypoxia induced non-coding transcriptome. Mol. Aspect. Med..

[7] Zou Z.W., Ma C., Medoro L., Chen L., Li P.D. (2016). LncRNA ANRIL is up-regulated in nasopharyngeal carcinoma and promotes the cancer progression via increasing proliferation, reprograming cell glucose metabolism and inducing side-population stem-like cancer cells. Oncotarget.

[8] Kang Y.H., Kim D., Jin E.J. (2015). Down-regulation of phospholipase D stimulates death of lung cancer cells involving up-regulation of the long ncRNA ANRIL. Anticancer Res..

[9] Lin Y., Zhao J., Wang H., Cao J., Nie Y. (2017 May). miR-181a modulates proliferation, migration and autophagy in AGS gastric cancer cells and downregulates MTMR3. Mol. Med. Rep..

[10] Tekirdag K.A., Korkmaz G., Ozturk D.G., Agami R., Gozuacik D. (2013). MIR181A regulates starvation- and rapamycin-induced autophagy through targeting of ATG5. Autophagy.

[11] Taguchi-atarashi N., Hamasaki M., Matsunaga K., Omori H., Ktistakis N.T. (2010). Modulation of local PtdIns3P levels by the PI phosphatase MTMR3 regulates constitutive autophagy. Traffic.

[12] Hao F., Itoh T., Morita E., Shirahama-Noda K., Yoshimori T. (2016). The PtdIns3-phosphatase MTMR3 interacts with mTORC1 and suppresses its activity. FEBS Lett..

[13] Ma Y., Wang P., Xue Y., Qu C., Zheng J., Liu X., Ma J., Liu Y. (2017 Mar). PVT1 affects growth of glioma microvascular endothelial cells by negatively regulating miR-186. Tumour Biol.

[14] Hao Y.R., Zhang D.J., Fu Z.M., Guo Y.Y., Guan G.F. (2019 Sep 20). Long non-coding RNA ANRIL promotes proliferation, clonogenicity, invasion and migration of laryngeal squamous cell carcinoma by regulating miR-181a/Snai2 axis. Regen Ther.

[15] Yin Yafei, Yang Wenqun, Zhang Lu, Liu Kang, Luo Zimian (2021). Long non-coding RNA ANRIL and its target microRNAs (microRNA-34a, microRNA-125a and microRNA-186) relate to risk stratification and prognosis in multiple myeloma. Hematology.

[16] Sisti G., Colombi I. (2019 Sep). New blood pressure cut off for preeclampsia definition: 130/80 mmHg. Eur. J. Obstet. Gynecol. Reprod. Biol..

[17] Livak K.J., Schmittgen T.D. (2001).

[18] Li Q., Zhang J., Su D.M., Guan L.N., Mu W.H., Yu M., Ma X., Yang R.J. (2020). lncRNA TUG1 modulates proliferation, apoptosis, invasion, and angiogenesis via targeting miR-29b in trophoblast cells. Hum. Genom..

[19] Zhao, Y.H.; Liu, Y.L.; Fei, K.L.; Li, P. Long non-coding RNA HOTAIR modulates the progression of preeclampsia through inhibiting miR-106 in an EZH2-dependent manner. Life Sci.. 253, 117668. ([CrossRef]). 10.1016/j.lfs.2020.117668 32320706

[20] Liu Z., Guo N., Zhang X.J. (2021). Long noncoding TUG1 promotes angiogenesis of HUVECs in PE via regulating the miR-29a- 3p/VEGFA and Ang2/Tie2 pathways. Microvasc. Res..

[21] Cirkovic A., Stanisavljevic D., Milin-Lazovic J., Rajovic N., Pavlovic V., Milicevic O. (2021). Preeclamptic women have disrupted placental microRNA expression at the time of preeclampsia diagnosis:meta analysis. Front. Bioeng. Biotechnol..

[22] Park J.W., Kim Y., Lee S.B., Oh C.W., Lee E.J., Ko J.Y., Park J.H. (2022 May). Autophagy inhibits cancer stemness in triple-negative breast cancer via miR-181a-mediated regulation of ATG5 and/or ATG2B. Mol. Oncol..

[23] Yang J., He Y., Zhai N., Ding S., Li J., Peng Z. (2018 Jan 1). MicroRNA-181a inhibits autophagy by targeting Atg5 in hepatocellular carcinoma. Front. Biosci..

[24] Wang L., Bi R., Li L., Zhou K., Yin H. (2021 Aug 10). lncRNA ANRIL aggravates the chemoresistance of pancreatic cancer cells to gemcitabine by targeting inhibition of miR-181a and targeting HMGB1-induced autophagy. Aging (Albany NY).

[25] Senousy M.A., Helmy H.S., Fathy N., Shaker O.G., Ayeldeen G.M. (2019 Aug 23). Association of MTMR3 rs12537 at miR-181a binding site with rheumatoid arthritis and systemic lupus erythematosus risk in Egyptian patients. Sci. Rep..

[26] Hao F., Itoh T., Morita E., Shirahama-Noda K., Yoshimori T. (2016). The PtdIns3-phosphatase MTMR3 interacts with mTORC1 and suppresses its activity. FEBS Lett..

[27] Dossou Akpedje S., Basu Alakananda (2019). The emerging roles of mTORC1 in macromanaging autophagy. Cancers.

[28] Taguchi-atarashi N., Hamasaki M., Matsunaga K., Omori H., Ktistakis N.T. (2010). Modulation of local PtdIns3P levels by the PI phosphatase MTMR3 regulates constitutive autophagy. Traffic.

[29] Han Y., Zhou S., Wang X., Mao E., Huang L. (2020 Jan). SNHG14 stimulates cell autophagy to facilitate cisplatin resistance of colorectal cancer by regulating miR-186/ATG14 axis. Biomed. Pharmacother..

[30] Zhang Y., Ma L., Lu E., Huang W. (2021 Apr 16). Atorvastatin upregulates microRNA-186 and inhibits the TLR4-mediated MAPKs/NF-κB pathway to relieve steroid-induced avascular necrosis of the femoral head. Front. Pharmacol..

[31] Bayoumi A.S., Sayed A., Broskova Z., Teoh J.P., Wilson J., Su H., Tang Y.L., Kim I.M. (2016). Crosstalk between long noncoding RNAs and MicroRNAs in health and disease. Int. J. Mol. Sci..

[32] Zhang J.J., Wang D.D., Du C.X., Wang Y. (2018). Long noncoding RNA ANRIL promotes cervical cancer development by acting as a sponge of miR-186. Oncology research.

[33] Xu Y., Chen J., Wang M., Yu R., Zou W., Shen W. (2021). Mechanism of lncRNA-ANRIL/miR-181b in autophagy of cardiomyocytes in mice with uremia by targeting ATG5. PLoS One.

[34] Lin Y., Zhao J., Wang H., Cao J., Nie Y. (2017). miR-181a modulates proliferation, migration and autophagy in AGS gastric cancer cells and downregulates MTMR3. Mol. Med. Rep..

